# A genetically encoded fluorescent biosensor for sensitive detection of cellular c-di-GMP levels in *Escherichia coli*

**DOI:** 10.3389/fchem.2024.1528626

**Published:** 2025-01-10

**Authors:** He Li, Shu Quan, Wei He

**Affiliations:** ^1^ Shanghai Frontiers Science Center of Optogenetic Techniques for Cell Metabolism, East China University of Science and Technology, Shanghai, China; ^2^ State Key Laboratory of Microbial Metabolism, School of Life Sciences and Biotechnology, Zhangjiang Institute for Advanced Study, Shanghai Jiao Tong University, Shanghai, China; ^3^ State Key Laboratory of Molecular Biology, Shanghai Institute of Biochemistry and Cell Biology, Center for Excellence in Molecular Cell Science, Chinese Academy of Sciences, Shanghai, China

**Keywords:** c-di-GMP, genetically encoded fluorescent biosensor, transcription factor, MrkH, diguanylate cyclases

## Abstract

Cyclic di-guanosine monophosphate (c-di-GMP) acts as a second messenger regulating bacterial behaviors including cell cycling, biofilm formation, adhesion, and virulence. Monitoring c-di-GMP levels is crucial for understanding these processes and designing inhibitors to combat biofilm-related antibiotic resistance. Here, we developed a genetically encoded biosensor, cdiGEBS, based on the transcriptional activity of the c-di-GMP-responsive transcription factor MrkH. Notably, cdiGEBS can detect both low and high cellular c-di-GMP levels, with a high fluorescence dynamic change of 23-fold. Moreover, it can detect subtle changes in c-di-GMP concentrations due to variations in the expression of c-di-GMP synthesis or degradation enzymes and can distinguish different synthesis activities among WspR mutants. These capabilities allow us to apply cdiGEBS for identifying new diguanylate cyclases and evaluating chemicals that modulate c-di-GMP levels, highlighting its potential as a high-throughput tool for screening inhibitors of c-di-GMP synthesis enzymes. Overall, cdiGEBS enhances the study of c-di-GMP-regulated functions and holds the potential for screening antimicrobials targeting c-di-GMP or its synthesis enzymes.

## Graphical Abstract

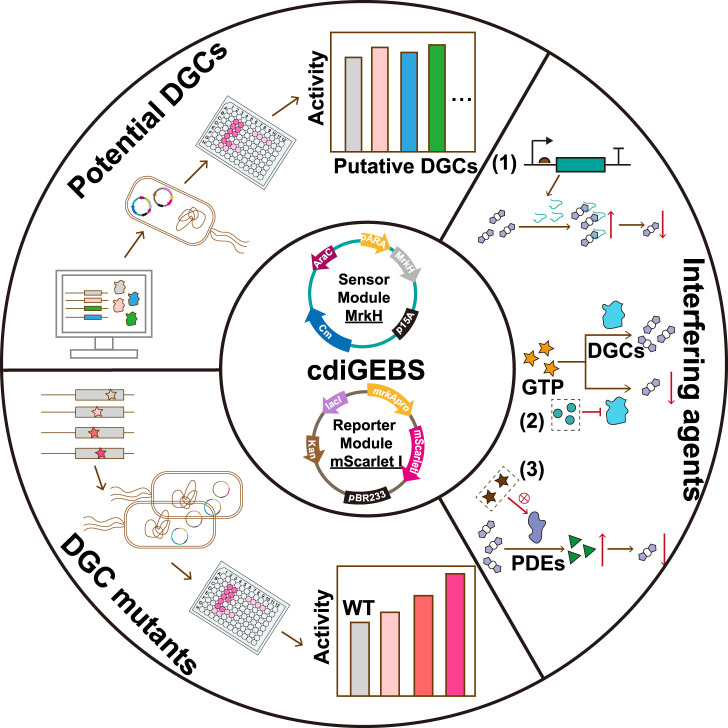

## 1 Introduction

C-di-GMP is a ubiquitous bacterial second messenger, regulating bacterial crucial behaviors and physiological functions, including biofilm, virulence, and the cell cycle (Römling et al., 2013; Ha and O’Toole, 2015; Kulasakara et al., 2006). Studies suggested that c-di-GMP plays a particularly pivotal role in governing the transition of bacteria from a planktonic state to a biofilm lifestyle (Jenal et al., 2017). Specifically, high c-di-GMP levels promote biofilm formation (Flemming et al., 2016), while low c-di-GMP levels could increase motility and facilitate biofilm dispersal (Rumbaugh and Sauer, 2020). Through biofilm formation, bacteria could defend against host immune responses and external environmental threats, including antimicrobials like colistin and chlorhexidine (Ciofu et al., 2015; Jenal et al., 2017; Wang Z. et al., 2023). To tightly regulate biofilm formation, bacteria have evolved sophisticated regulatory networks that maintain c-di-GMP homeostasis (Jenal and Malone, 2006; Park and Sauer, 2022).

Intracellular c-di-GMP levels are predominantly regulated by two types of antagonistic enzymes, diguanylate cyclases (DGCs) and phosphodiesterases (PDEs). DGCs that possess the GG(D/E)EF domain synthesize c-di-GMP by cyclizing two molecules of guanosine triphosphate (GTP) (Schirmer, 2016). In contrast, PDEs featuring either EAL or HD-GYP domains, catalyze the linearization of c-di-GMP into 5′-phosphoguanylyl-(3′-5′)-guanosine (pGpG) or degrade c-di-GMP into two molecules of guanosine monophosphate (GMP) (Christen et al., 2005; Bellini et al., 2014). The c-di-GMP signaling network is characterized by numerous DGCs and PDEs, many of which contain putative sensory domains that perceive environmental cues (Almblad et al., 2021; Randall et al., 2022).

In order to gain deeper insights into c-di-GMP-mediated bacterial physiological functions and explore uncharacterized c-di-GMP signaling regulatory pathways, numerous tools have been developed to monitor intracellular c-di-GMP fluctuations (Petchiappan et al., 2020; McCaughey et al., 2024). Liquid chromatography coupled with gas chromatography-mass spectrometry (LC-GC/MS) is frequently employed to quantify c-di-GMP concentrations in extracted biological samples due to high accuracy and sensitivity (Simm et al., 2009; Irie and Parsek, 2014). However, the complex sample preparation and costly equipment required for these analytical tools limit their application. To overcome these limitations, genetically encoded fluorescent biosensors, typically incorporated into cells as plasmid DNA, have been extensively developed for monitoring the spatiotemporal dynamics of signaling molecules in living cells (Palmer et al., 2011; Greenwald et al., 2018; Wang et al., 2022). Specifically, for c-di-GMP detection *in vivo*, a serial of genetically encoded fluorescent biosensors have been established, including the fluorescence resonance energy transfer (FRET) - based biosensors (Christen et al., 2010; Petersen et al., 2019), the bimolecular fluorescence complementation (BiFC) - based biosensors (Halte et al., 2022), the sensor protein conformational change-based biosensors and others (Ho et al., 2013; Vrabioiu and Berg, 2022; Kaczmarczyk et al., 2024; McCaughey et al., 2024).

These biosensors have significantly advanced the study of c-di-GMP-mediated transcriptional pathways and bacterial behaviors. However, since these sensors typically require direct coupling of the c-di-GMP responsive element with a fluorescent reporter, amplifying the fluorescent signal and achieving a high fluorescence dynamic range remains challenging. In contrast, transcription factor (TF) - based biosensors, which rely on the transcriptional activity of the TF, can amplify the detection signal. These biosensors typically comprise a TF and a TF-recognized promoter fused to a fluorescent protein-encoding gene. Upon binding to the target molecule, the TF’s transcriptional activity is activated, leading to robust expression of the fluorescent protein reporter. Rybtke et al. developed a TF activity-based *pCdrA::gfp* reporter for c-di-GMP detection in *Pseudomonas aeruginosa*, significantly advancing the study of c-di-GMP-regulated cellular processes in this species (Rodesney et al., 2017; Wang L. et al., 2023; Hendrix et al., 2024; Kennelly et al., 2024). However, such a straightforward set up utilizing the ligand-regulated transcriptional activity of TF has not been established in *E. coli*. Current TF-based biosensors in *E. coli* rely on conformational changes in the TF upon c-di-GMP binding, as in the mScarlet I-mVenus^NB^-MrkH biosensor (Vrabioiu and Berg, 2022), or on TF dimerization induced by c-di-GMP binding, as in the BiFC-based CensYBL biosensor (Halte et al., 2022), resulting in a relatively limited dynamic range of fluorescence for c-di-GMP detection (0.9 to 3-fold).

In this study, we introduced a TF transcriptional activity-based c-di-GMP biosensor (named cdiGEBS for c-di-GMP Genetically Encoded BioSensor), which utilized the c-di-GMP-responsive transcription factor MrkH from *Klebsiella pneumonia* and its corresponding promoter (P*mrkA*) as the sensing module, with the fluorescent protein mScarlet I as the reporter element. Our sensor demonstrates a fluorescence dynamic range of 23-fold, substantially improving the sensitivity for detecting both elevated and reduced intracellular c-di-GMP levels. Notably, cdiGEBS is capable of detecting subtle changes in c-di-GMP concentrations resulting from altered expression of its biogenesis or degradation enzymes. Additionally, it can distinguish the c-di-GMP synthesis activities of different mutants of the diguanylate cyclase WspR. These features encourage us to successfully apply cdiGEBS for identifying novel diguanylate cyclases and assessing the impact of various compounds on cellular c-di-GMP levels. Taken together, cdiGEBS not only serves as a versatile and easy-to-use tool for the functional analysis of c-di-GMP biogenesis enzymes but also shows promise for high-throughput screening of novel inhibitors targeting c-di-GMP synthesis for combating biofilm-associated bacterial infections.

## 2 Materials and methods

### 2.1 Plasmid constructions

Strains and plasmids used in this paper are listed in Table 1. Plasmid construction in this work follows the overlap extension PCR cloning procedure as described previously (Nelson and Fitch, 2011). *E. coli* strain Turbo (Tsingke Biotech) was used for competent cell preparation. The *wspR*, *mrkA* promoter, and *mrkH* sequences were synthesized by Tsingke Biotech, while the *dgcT*, *dgcI*, *pdeH*, and *dgcF* sequences were amplified from the genome of the *E. coli* MG1655 strain and the *proC* promoter and *mScarlet I* sequences were amplified from pEB2-mScarlet I-mVenus^NB^-MrkH vector (Addgene No. 182291).

**TABLE 1:** Strains and plasmids used in this work.

Strains and plasmids	Genotype and relevant description	Source or reference no.
*E.coli* strains		
Turbo	*F* ^*−*^ *[proA* ^*+*^ *B* ^*+*^ *lacIq ΔlacZM15] fhuA Δ(lac-proAB)glnV galE15 galK16*)	Lab stock
XZX118	*F* ^*−*^ *lambda* ^*-*^ *ilvG* ^*-*^ *rfb* ^*-*^ *50 rph-1 ΔhsdR ΔampC lacZ::T7p07 ΔaraBAD*	Lab stock
Plasmids		
pET28b(+)-P*mrkA*-mScarlet I	Kan^r^; the reporter plasmid of cdiGEBS	This study
pBAD33-MrkH	Cm^r^; for inducible expression of MrkH in Figure 1C	This study
pBAD33-MrkH R113A	Cm^r^; for inducible expression of MrkH R113A in Figure 1C	This study
pBAD33-MrkH-WspR D70E	Cm^r^; for inducible expression of MrkH and WspR D70E in Figure 1C	This study
pBAD33-MrkH-PdeH	Cm^r^; for inducible expression of MrkH and PdeH in Figure 1C	This study
pBAD33-6 × His-MrkH	Cm^r^; for inducible expression of 6 × His-MrkH, used in Western blot experiments in Supplementary Figure S2	This study
pBAD33-6 × His-MrkH-WspR D70E	Cm^r^; for inducible expression of 6 × His-MrkH and WspR D70E, used in Western blot experiments in Supplementary Figure S2	This study
pBAD33-6 × His-MrkH-PdeH	Cm^r^; for inducible expression of 6 × His-MrkH and PdeH, used in Western blot experiments in Supplementary Figure S2	This study
pBAD33-6 × His-MrkH R113A	Cm^r^; for inducible expression of 6 × His-MrkH R113A, used in Western blot experiments in Supplementary Figure S2	This study
pBAD33-WspR D70E-*proC*-MrkH	Cm^r^; for constitutive expression of MrkH and inducible expression of WspR D70E, used in the sensitivity characterization experiment of cdiGEBS in Figure 2B	This study
pBAD33-PdeH-*proC*-MrkH	Cm^r^; for constitutive expression of MrkH and inducible expression of PdeH, respectively; used in the sensitivity characterization experiment of cdiGEBS in Figure 2C	This study
pBAD33-*proC*-MrkH	Cm^r^; for constitutive expression of MrkH, used in the sensitivity characterization experiment of cdiGEBS in Supplementary Figure S3	This study
pBAD33-MrkH-DgcT	Cm^r^; for inducible expression of MrkH and DgcT, used in the diguanylate cyclase activity detection assay in Figure 3B	This study
pBAD33-MrkH-DgcI	Cm^r^; for inducible expression of MrkH and DgcI, used in the diguanylate cyclase activity detection assay in Figure 3B	This study
pBAD33-MrkH-DgcF	Cm^r^; for inducible expression of MrkH and DgcF, used in the diguanylate cyclase activity detection assay in Figure 3B	This study
pBAD33-MrkH-DgcT E361A	Cm^r^; for inducible expression of MrkH and DgcT E361A, used in the diguanylate cyclase activity detection assay in Figure 3B	This study
pBAD33-MrkH-DgcI D371A	Cm^r^; for inducible expression of MrkH and DgcI D371A, used in the diguanylate cyclase activity detection assay in Figure 3B	This study
pBAD33-MrkH-DgcF E224A	Cm^r^; for inducible expression of MrkH and DgcF E224A, used in the diguanylate cyclase activity detection assay in Figure 3B	This study
pBAD33-MrkH-WspR	Cm^r^; for inducible expression of MrkH and WspR in Figure 3B	This study
pBAD33-MrkH-WspR D70A	Cm^r^; for inducible expression of MrkH and WspR D70A in Figure 3B	This study
pBAD33-MrkH-WspR E253A	Cm^r^; for inducible expression of MrkH and WspR E253A in Figure 3B	This study
pBAD33-MrkH-WspR D70E	Cm^r^; for inducible expression of MrkH and WspR D70E in Figure 3B	This study
pBAD33-MrkH-WspR L170D	Cm^r^; for inducible expression of MrkH and WspR L170D in Figure 3B	This study
pBAD33-MrkH-WspR V72D	Cm^r^; for inducible expression of MrkH and WspR V72D in Figure 3B	This study
pBAD33-6 × His-MrkH-6 × His-WspR	Cm^r^; for inducible expression of 6 × His MrkH and 6 × His-WspR, used in Western blot experiments in Supplementary Figure S4B	This study
pBAD33-6 × His-MrkH-6 × His-WspR L170D	Cm^r^; for inducible expression of 6 × His MrkH and 6 × His-WspR L170D, used in Western blot experiments in Supplementary Figure S4B	This study
pBAD33-6 × His-MrkH-6 × His-WspR E253A	Cm^r^; for inducible expression of 6 × His MrkH and 6 × His-WspR E253A, used in Western blot experiments in Supplementary Figure S4	This study
pBAD33-6 × His-MrkH-6 × His-WspR D70E	Cm^r^; for inducible expression of 6 × His MrkH and 6 × His-WspR D70E, used in Western blot experiments in Supplementary Figure S4	This study
pBAD33-6 × His-MrkH-6 × His-WspR V72D	Cm^r^; for inducible expression of 6 × His MrkH and WspR V72D, used in Western blot experiments in Supplementary Figure S4	This study
pCDFDuet-6 × His-SUMO-CSP2	Strep^r^ and Spec^r^; for inducible expression of 6 × His-SUMO-CSP2 in Figures 4B, C	This study
pCDFDuet-6 × His-SUMO-CSP2 R169A	Strep^r^ and Spec^r^; for inducible expression of 6 × His-SUMO-CSP2 R169A in Figures 4B, C	This study
pCDFDuet-6 × His-SUMO-CSP3	Strep^r^ and Spec^r^; for inducible expression of 6 × His-SUMO-CSP3 in Figures 4B, C	This study
pCDFDuet-Empty	Strep^r^ and Spec^r^; used as a negative control in Figures 4B, C	Lab stock

The *mrkH* gene was inserted into the medium-copy plasmid pBAD33, generating the plasmid pBAD33-MrkH. Subsequently, the *pdeH* or *wspR D70E* gene, along with an additional ribosome binding site, was inserted downstream of the *mrkH* gene, resulting in the plasmids pBAD33-MrkH-PdeH and pBAD33-MrkH-WspR D70E, respectively. Plasmid pET28b(+)-P*mrkA*-mScarlet I was constructed by replacing the *T7* promoter of the high-copy plasmid pET28b(+)-MCS with *mrkA* promoter and mScarlet I-encoding gene. For the plasmids pBAD33-PdeH-*proC*-MrkH and pBAD33-WspR D70E-*proC*-MrkH, which express PdeH or WspR D70E under the control of the *araBAD* promoter and constitutively express MrkH via the *proC* promoter respectively, were constructed by first inserting the PdeH or WspR D70E gene downstream of the *araBAD* promoter, followed by the insertion of the *proC* promoter, an additional ribosome binding site, and *mrkH* gene downstream of the *pdeH* or *wspR D70E* gene.

Quick change mutagenesis method was employed for introducing point mutations of the *wspR*, *dgcT*, *dgcI*, and *dgcF* genes (Liu and Naismith, 2008). All the oligonucleotides, promoters, and genes used in this work are listed in Supplementary Tables S1, S2.

### 2.2 Fluorescence microscopy

Overnight cultures of the strains harboring cdiGEBS and either PdeH or WspR D70E were diluted 1:100 into 4 mL of fresh LB medium supplemented with 100 μg mL^−1^ kanamycin, 34 μg mL^−1^ chloramphenicol, and 0.002% L-arabinose. Cells were then cultivated at 30°C for 6 h with a shaking speed of 220 rpm. Subsequently, 0.1 OD_600 nm_ cells were collected and resuspended in 40 µL phosphate-buffered saline (PBS). A 5 µL aliquot was placed on a microscope slide, coated with 1% agarose, and covered with a coverslip. Fluorescence images were captured using a Zeiss LSM 880 Confocal Laser Scanning Microscope, with the excitation wavelength set to 568 nm.

### 2.3 Fluorescence measurement with a microplate reader

Overnight cultures of *E. coli* co-expressing cdiGEBS and PdeH, or cdiGEBS and each WspR mutant, or cdiGEBS and each putative DGC were inoculated at a 1:100 dilution-fold into 4 mL fresh LB medium supplemented with 100 μg mL^−1^ kanamycin, 34 μg mL^−1^ chloramphenicol, and 0.002% L-arabinose. After grown at 30°C for 6 h, 1 OD_600 nm_ cells were collected and resuspended in 220 µL PBS for fluorescence measurements by an automated microplate reader (SynergyHTX hybrid reader, BioTek) with the excitation and the emission set at 540 ± 35 nm and 600 ± 40 nm, respectively.

### 2.4 Fluorescence intensity and fluorescence spectra detection by a fluorescence spectrometer

Overnight cultures of *E. coli* co-expressing cdiGEBS and various CSPs were inoculated at a 1:100 dilution-fold into 4 mL fresh LB medium supplemented with 100 μg mL^−1^ kanamycin, 34 μg mL^−1^ chloramphenicol, 100 μg mL^−1^ streptomycin, 100 μg mL^−1^ spectinomycin, and 0.002% L-arabinose. Cells were then cultured at 30°C, 220 rpm until the OD_600 nm_ reached 0.3, followed by adding 0.1 mM isopropyl-β-D-thiogalactoside (IPTG) to induce the expression of 6 × His-MBP-CSP. The 6 × His-MBP tag was fused with various CSP to prevent cellular degradation of CSP (Hee et al., 2020). Following 6 h of 6 × His-MBP-CSP expression, cells equivalent to 4 OD_600 nm_ units were harvested and resuspended in 2 mL of PBS buffer for fluorescence intensity and spectral measurements using a fluorescence spectrometer (FS5 Spectrofluorometer, Edinburgh Instruments), with the excitation wavelength set at 568 nm and an emission range of 575–650 nm.

### 2.5 Time-dependent fluorescence detection

Overnight cultures of *E. coli* harboring cdiGEBS were diluted 1:100 into fresh LB medium supplemented with 100 μg mL^−1^ kanamycin, 34 μg mL^−1^ chloramphenicol, and 0.002% L-arabinose. Then 200 μL cell cultures were transferred to each well of a 96-well plate containing different concentrations of tyrosol or ampicillin. Fluorescence intensity and cell density (OD_600 nm_) were monitored by using a Synergy HTX Multi-Mode Microplate Reader with a 15-min detection period for 21 h at 30°C. For fluorescence intensity measurement, the excitation and emission wavelength were set at 540 ± 35 nm and 600 ± 40 nm, respectively.

### 2.6 Western blots analysis

Cells were cultivated and induced for protein expression as described in the “Fluorescence Measurement by a Microplate Reader” Section. 1 OD_600 nm_ cells were collected and resuspended in PBS to a final OD_600 nm_ of 5.0. Then cell samples were mixed with 5 × protein loading buffer, and subjected to 12% SDS-PAGE after boiling for 10 min. For Western blot analysis, proteins were transferred to polyvinylidene fluoride (PVDF) membranes (Merck) using a wet transfer apparatus (Tanon). Then the PVDF membranes were blocked with 5% non-fat milk in TBST (Tris buffer saline containing 0.1% Tween-20), followed by incubation with the primary antibodies: anti-6 × His from mouse or anti-trigger factor from rabbit (1:4,000, GenScript, Cat. No. A01329) in TBST containing 5% non-fat milk. Afterward, the membranes were incubated with fluorescently labeled IRDye 800 CW secondary antibodies (1:10,000, LI-COR Biosciences), and fluorescence images were recorded using the Odyssey Sa Infrared Imaging System (LI-COR Biosciences).

## 3 Results

### 3.1 Construction and characterization of a transcription factor-based c-di-GMP biosensor

**FIGURE 1:**
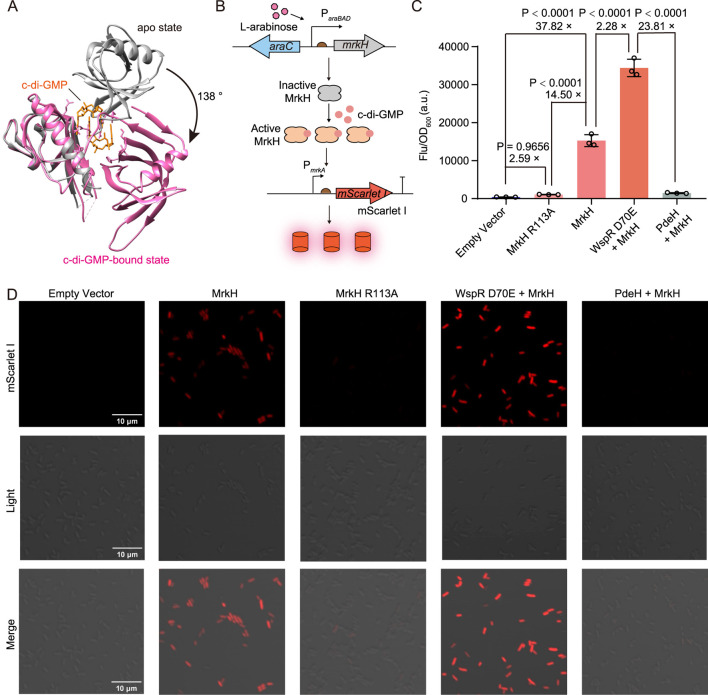
Construction and characterization of cdiGEBS. **(A)** Structural alignment of the apo (gray, PDB: 5KEC) and c-di-GMP-bound MrkH (pink, PDB: 5KGO). **(B)** Schematic illustration of the working mechanism of cdiGEBS. **(C)** Performance of cdiGEBS in detecting both high and low cellular c-di-GMP levels. Fluorescence was measured in strains co-harboring the P*mrkA*-mScarlet I plasmid and the pBAD33 plasmid expressing the indicated proteins. An additional ribosome binding site, fused with the gene encoding WspR D70E or PdeH, was cloned downstream of the *mrkH* sequence on the pBAD33 plasmid to enable co-expression of WspR D70E or PdeH with MrkH. The strain harboring both pBAD33-Empty and P*mrkA*-mScarlet I plasmids served as the “Empty vector” control. Samples were induced with 0.002% L-arabinose at 30°C for 6 h. Fluorescence was recorded by using a microplate reader with the excitation and emission wavelengths set at 540 ± 35 nm and 600 ± 40 nm, respectively. Individual data points (circles) and mean ± SD (n = 3) are shown. **(D)** Fluorescence microscopy images of strains expressing the corresponding proteins. Statistical analysis was performed with GraphPad Prism 8 using one-way ANOVA with Tukey’s multiple comparisons tests.

The transcriptional regulator MrkH is a member of the PliZ family and exhibits a high affinity for c-di-GMP in the nanomolar range (Schumacher and Zeng, 2016; Wang F. et al., 2016). Upon c-di-GMP binding, MrkH undergoes a conformational change-induced activation (Figure 1A) (Schumacher and Zeng, 2016), allowing it to bind the *mrkA* promoter and highly upregulate the transcription of the downstream *mrkABCDF* operon (Wilksch et al., 2011). Based on this, we introduced the c-di-GMP sensing element MrkH under the regulation of the *araBAD* promoter and the fluorescent reporter element mScarlet I under the control of the *mrkA* promoter in *E. coli* to establish a genetically encoded fluorescent biosensor for c-di-GMP detection. Specifically, the *mrkH* gene was placed downstream of the *araBAD* promoter in the medium-copy number plasmid pBAD33 vector, while the mScarlet I gene was inserted downstream of the *mrkA* promoter (P*mrkA*) on the high-copy plasmid pET28b(+) backbone with the original *T7* promoter removed (Figure 1B).

We observed a significant fluorescence increase when the strain containing the biosensor was induced with arabinose for MrkH expression, suggesting that the endogenous c-di-GMP in *E. coli* can substantially activate the transcriptional activity of MrkH and thus upregulate mScarlet I expression (Figure 1C). To further confirm that the observed increase in mScarlet I fluorescence was due to the transcriptional activation of MrkH upon binding c-di-GMP, we introduced the R113A mutation in MrkH to substantially diminish its c-di-GMP-binding ability (Vrabioiu and Berg, 2022). We found that the resultant strain expressing the MrkH R113A mutant exhibited a significantly decreased fluorescence intensity compared to the strain expressing the wild-type MrkH (Figure 1C), indicating that the fluorescence increase in the strain expressing wild-type MrkH was indeed mediated by the binding between MrkH and c-di-GMP.

To assess whether cdiGEBS can respond to varying intracellular levels of c-di-GMP, we co-expressed the biosensor with either a constitutively activated diguanylate cyclase WspR D70E or the phosphodiesterase PdeH to induce high or low c-di-GMP concentrations, respectively (Dippel et al., 2018). By optimizing the arabinose concentration, cultivation temperature, and induction time, we found that the biosensor could effectively distinguish between high and low c-di-GMP levels, exhibiting a remarkable fluorescence change readout of over 23-fold (Supplementary Figure S1 and Figure 1C). These findings were further supported by fluorescence imaging (Figure 1D), and Western blot analysis confirmed that the observed differences in fluorescence intensity were not due to variations in MrkH expression levels (Supplementary Figure S2). Taken together, these results indicate that cdiGEBS can respond to high and low c-di-GMP concentrations with highly dynamic fluorescence fold changes.

### 3.2 Distinguishing various cellular c-di-GMP levels with cdiGEBS

**FIGURE 2:**
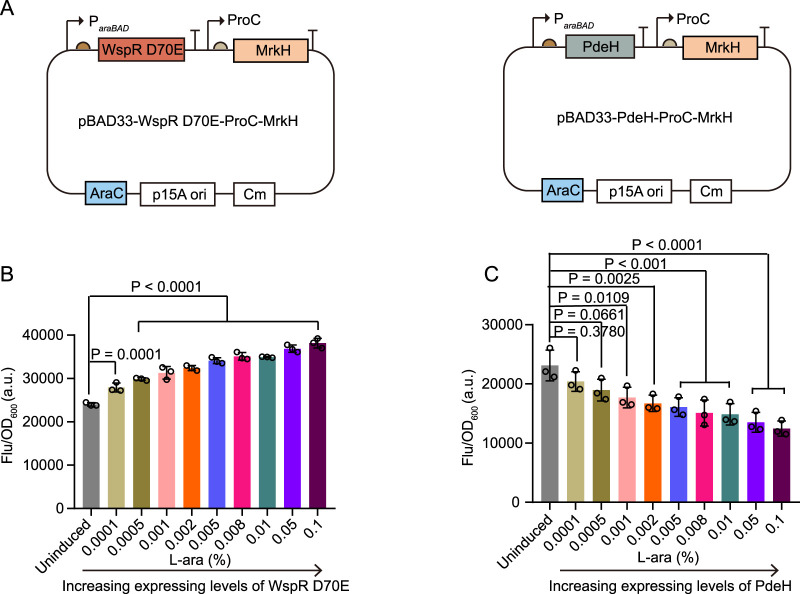
Sensitivity characterization of cdiGEBS. **(A)** Schematic illustrations of the modified biosensors for sensitivity characterization. **(B, C)** Normalized mScarlet I fluorescence intensities (Ex = 540 ± 35 nm, Em = 600 ± 40 nm, measured with a microplate reader) of strains co-expressing cdiGEBS with WspR D70E **(B)** or PdeH **(C)** under increasing concentrations of L-arabinose. Overnight cultures were inoculated into fresh LB culture at a 1:100 dilution fold. At the same time, various concentrations of L-arabinose were added to induce WspR D70E or PdeH expression at 30°C for 9 h. Subsequently, 1 OD cells were collected and used for fluorescence measurement. Individual data points (circles) and mean ± SD (n = 3) are shown. Statistical analysis was performed with GraphPad Prism 8 using one-way ANOVA with Tukey’s multiple comparisons tests.

To further investigate the sensitivity of cdiGEBS in distinguishing varying cellular c-di-GMP levels, we maintained the mScarlet I reporter element and modified the sensing element as follows: 1) MrkH was constitutively expressed under the control of the *proC* promoter, and 2) WspR D70E or PdeH expression was induced by the *araBAD* promoter (Figure 2A). By titrating the resulting strains with different concentrations of L-arabinose, we were able to gradually adjust the levels of the c-di-GMP synthase WspR D70E or the degradation enzyme PdeH, leading to progressively increased or decreased cellular c-di-GMP concentrations, respectively.

We observed that the strain co-expressing MrkH and WspR D70E exhibited a gradual increase in mScarlet I fluorescence with increasing concentrations of L-arabinose (Figure 2B). In contrast, the strain co-expressing MrkH and PdeH showed a dose-dependent decrease in mScarlet I fluorescence intensity with L-arabinose (Figure 2C). Furthermore, by titrating the strain containing only the biosensor, without WspR D70E or PdeH, with different concentrations of L-arabinose, we excluded the possibility that these fluorescence changes were due to the effects of L-arabinose on the mScarlet I fluorophore or MrkH transcriptional activity (Supplementary Figure S3). Overall, these results demonstrate that cdiGEBS is sensitive and capable of distinguishing subtle variations in intracellular c-di-GMP levels, which result from finely tuned adjustments in the expression of c-di-GMP synthesis or degradation enzymes.

### 3.3 Assessment of diguanylate cyclase activities *in vivo*

**FIGURE 3:**
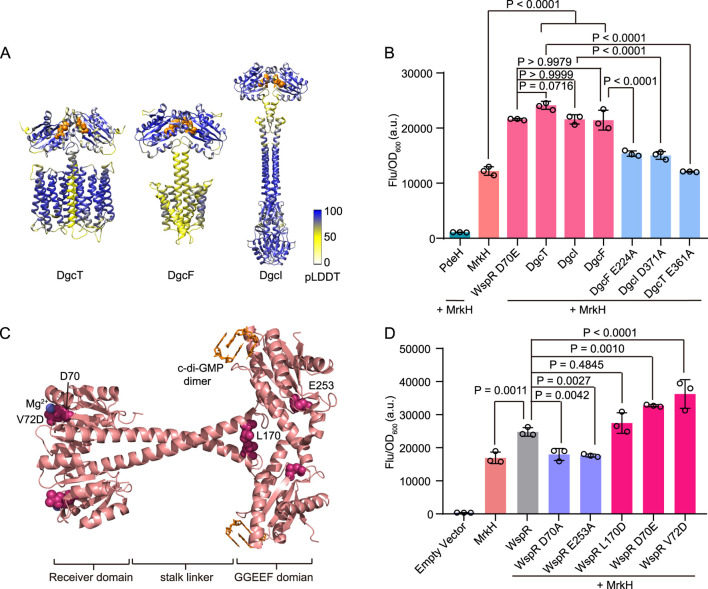
Diguanylate cyclase activity detection using cdiGEBS. **(A)** Homodimer structure prediction of DgcT, DgcF, and DgcI by AlphaFold 3. The white-to-blue color scale indicates pLDDT scores ranging from 0 to 100. The GG(D/E)EF domain of each structure was represented as orange spheres. **(B)** Diguanylate cyclase activity assessment of wild-type and mutants of the putative DGCs using cdiGEBS. All strains contain the P*mrkA*-mScarlet I reporter plasmid. PdeH, WspR D70E, DgcT, DgcI, DgcF, DgcT E361A, DgcI D371A, or DgcF E224A was co-expressed with MrkH under the control of the *araBAD* promoter on the pBAD33 plasmid. Samples were induced with 0.002% L-arabinose at 30°C for 6 h **(C)** WspR dimer structure (PDB: 3BRE) with each mutation site represented as a pink sphere. C-di-GMP dimer was colored orange and the magnesium ion was colored blue. **(D)** Distinguishing the activity differences of various WspR mutants using cdiGEBS. Wild-type WspR and its mutants were co-expressed with MrkH under the control of the *araBAD* promoter on the pBAD33 plasmid. The strain harboring both pBAD33-Empty and P*mrkA*-mScarlet I plasmids was served as the “Empty vector” control. For **(B)** and **(D)**, samples were excited at 540 ± 35 nm and the fluorescence signals were recorded at 600 ± 40 nm by using a microplate reader. Individual data points (circles) and mean ± SD (n = 3) are shown. Statistical analysis was performed with GraphPad Prism 8 using one-way ANOVA with Tukey’s multiple comparisons tests.

The capability of cdiGEBS to distinguish varying c-di-GMP levels promoted us to explore its application in assessing the activity of diguanylate cyclases (DGCs). There are 29 genes in *Escherichia coli* K-12 participating in c-di-GMP biosynthesis or degradation (Hengge et al., 2016; Povolotsky and Hengge, 2016). Previous studies have shown that overexpression of DgcT resulted in a significant increase of intracellular c-di-GMP, implying that DgcT processed diguanylate cyclase activity (Jonas et al., 2008). Additionally, the uncharacterized membrane-associated proteins DgcI and DgcF have also been annotated as putative DGCs in *E. coli* since strains lacking DgcF or DgcI (*ΔdgcF* or *ΔdgcI*) display increased swimming motility (Sanchez-Torres et al., 2011; Povolotsky and Hengge, 2016). DGCs generally function as homodimers, with their GG(D/E)EF domains being responsible for the production of c-di-GMP (Dahlstrom and O’Toole, 2017). To gain structural insights into these putative DGCs, we predicted the potential dimeric structures of these proteins using AlphaFold 3 and identified the presence of typical GG(D/E)EF domains in all of them (Figure 3A) (Abramson et al., 2024).

We then investigated the *in vivo* diguanylate cyclase activities of these putative DGCs using cdiGEBS, with the constitutively active DGC WspR D70E serving as the positive control. Strains co-expressing the biosensor and any of the putative DGCs showed a substantial increase in mScarlet I fluorescence intensity, comparable to the strains co-expressing the biosensor and WspR D70E (Figure 3B). This result indicates that these three putative DGCs can indeed catalyze the synthesis of c-di-GMP *in vivo*. Moreover, to investigate the effect of the GG(D/E)EF domain of DgcT, DgcF, and DgcI on their DGC activity, we substituted the third residue of the GG(D/E)EF domain with alanine. We found that the resulting strains expressing the protein mutants (DgcT E361A, DgcI D371A, and DgcF E224A) showed significantly decreased fluorescence intensities compared to the strains expressing the wild-type proteins (Figure 3B). This suggests that DgcT, DgcI, and DgcF all catalyze the synthesis of c-di-GMP in a GG(D/E)EF domain-dependent manner.

The sensitive performance of our biosensor in tracking DGC activities drove us to further investigate whether it could discriminate the subtle differences in the enzymatic activities of various DGC mutants. To examine this, we characterized the activities of the canonical DGC WspR and its mutants with defined DGC activities *in vivo* using cdiGEBS (Figures 3C, D) (Huangyutitham et al., 2013). The D70A substitution disrupts the phosphorylation site, resulting in the loss of cyclase activity (De et al., 2009). Similarly, the E253A mutation inactivates WspR by impairing the active site (Malone et al., 2007). In contrast, the D70E mutation mimics the phosphorylated state, leading to constitutive activation (Wang X. C. et al., 2016). The V72D mutant also exhibits constitutive diguanylate cyclase activity, as identified in a random mutagenesis study of WspR (Huangyutitham et al., 2013). Additionally, studies have demonstrated that the tetrameric form of WspR displays high activity levels, with the WspR L170D variant enhancing activity by stabilizing this tetrameric form (De et al., 2008).

We found that strains co-expressing the biosensor and the wild-type WspR showed a significant increase in fluorescence intensity compared with the strain containing only the biosensor. In contrast, strains co-expressing the biosensor and the inactive mutants WspR D70A or WspR E253A exhibited low fluorescence intensities similar to the strain harboring the biosensor only, implying that these mutations nearly completely abolished DGC activities (Figure 3D). Consistent with the literature, we observed that strains co-expressing the biosensor and activity-enhanced mutants D70E, V72D, and L170D all showed a trend of increased fluorescence intensity compared to the strain co-expressing the biosensor and the wild-type WspR, although the increase in fluorescence intensity of the strain expressing the mutant L170D was not statistically significant (Figure 3D) (Huangyutitham et al., 2013). Additionally, Western blot analysis suggested that the observed fluorescence differences were not due to variations in the expression levels of MrkH and WspR mutants (Supplementary Figure S4). Taken together, these results suggested that cdiGEBS was sensitive and could be used for functional analysis and activity evaluation of DGCs *in vivo*.

### 3.4 Monitoring the interference of chemicals on the c-di-GMP levels *in vivo*

High c-di-GMP levels could stimulate biofilm formation, leading to a 10 to 1,000-fold increase in antibiotic resistance of bacteria in the sessile state than in the planktonic state (Dostert et al., 2019; Ciofu and Tolker-Nielsen, 2019; Dostert et al., 2021). Decreasing the cellular c-di-GMP levels is a promising strategy for combating biofilm-related antibiotic resistance and potential chronic infection. Therefore, researchers have developed a number of structurally diverse compounds to diminish c-di-GMP concentrations in bacteria, either by directly sequestering c-di-GMP or by enhancing PDE activity (Qvortrup et al., 2019; Hee et al., 2020). Encouraged by the sensitivity of cdiGEBS in monitoring c-di-GMP levels *in vivo*, we next sought to examine whether this sensor was capable of reflecting the effects of typical anti-biofilm chemicals on the cellular c-di-GMP level.

**FIGURE 4:**
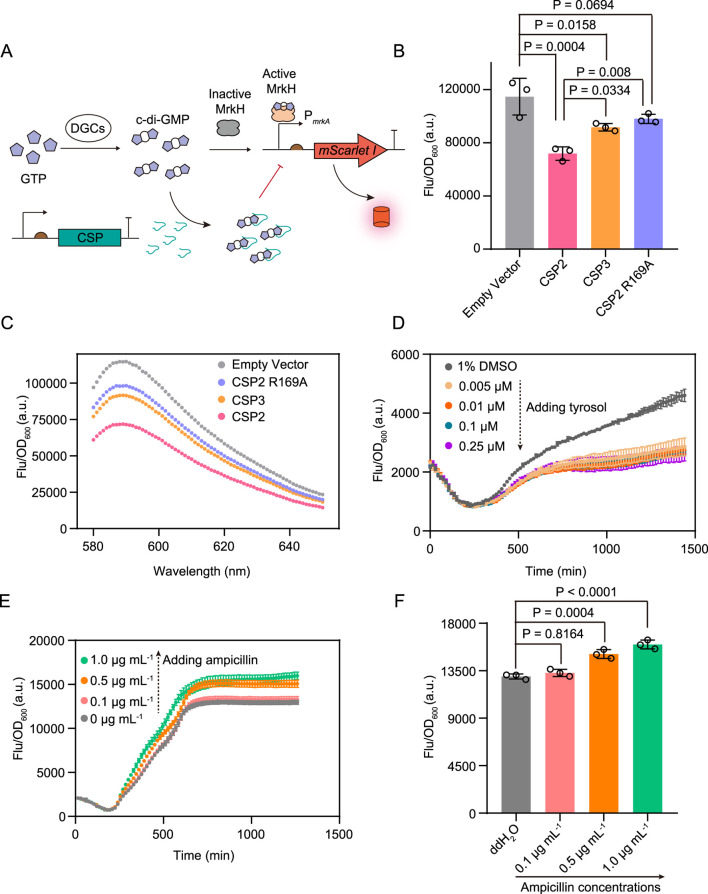
The application of cdiGEBS for investigating the effects of chemical compounds on cellular c-di-GMP levels. **(A)** Schematic illustration for detecting the effects of CSPs on cellular c-di-GMP levels by cdiGEBS. **(B)** Fluorescence intensities of strains co-expressing different CSPs and cdiGEBS. pCDFDuet vectors expressing each CSP were co-transformed with the cdiGEBS plasmids (pBAD33-MrkH, P*mrkA*-mScarlet I). The strain harboring the pBAD33-MrkH, P*mrkA*-mScarlet I, and pCDFDuet-Empty plasmids were used as the “Empty vector” control. **(C)** Fluorescence emission spectra of strains co-expressing different CSPs and cdiGEBS. Representative curves from three independent measurements are shown. For **(B, C)**, fluorescence was recorded by using a fluorescence spectrometer with the excitation and emission wavelengths set at 568 nm and 589 nm, respectively. **(D, E)** Time-dependent fluorescence changes of strains expressing cdiGEBS in the presence of various concentrations of tyrosol **(D)** and ampicillin **(E)**. Strains expressing cdiGEBS were cultured in 96-well microplates to allow continuous measurement of fluorescence and optical density using a microplate reader. **(F)** Fluorescence intensities of strains expressing cdiGEBS after 21 h of treatment with various concentrations of ampicillin. For **(B, F)**, individual data points (circles) and mean ± SD (*n* = 3) are shown. Statistical analysis was performed with GraphPad Prism 8 using one-way ANOVA with Tukey’s multiple comparisons tests.

Hee et al. designed a serial of short c-di-GMP-sequestering peptides (CSP) which could directly bind c-di-GMP and thus reduce the concentration of free cellular c-di-GMP in *Pseudomonas aeruginosa* and consequently inhibit biofilm formation (Hee et al., 2020). We found that the strain co-expressing the biosensor and the peptide CSP2 which bound c-di-GMP tightest exhibited a substantial decrease in mScarlet I fluorescence intensity compared with the strain harboring the biosensor only (Figures 4A–C). In contrast, co-expressing the biosensor and the binding affinity attenuated peptides CSP3 or CSP2 R169A only led to a moderate fluorescence decrease of the resulting strains (Figures 4B, C).

Furthermore, Choi et al. demonstrated that tyrosol could decrease c-di-GMP levels by activating PDE activity (Choi and Kim, 2024). We observed that treating the strain containing cdiGEBS with increasing concentrations of tyrosol resulted in decreased mScarlet I fluorescence intensity compared with the control strain which was treated with 1% DMSO (Figure 4D). In contrast to biofilm dispersal chemicals, studies have shown that subinhibitory concentrations of ampicillin can facilitate c-di-GMP production (Boehm et al., 2009; Ho et al., 2013; Gao et al., 2022). We also found that cdiGEBS could be used for monitoring the increasing effect of ampicillin on the cellular c-di-GMP levels (Figure 4F). Additionally, neither over-expressing the CSPs nor tyrosol treatment substantially hinders bacterial growth (Supplementary Figure S5). For ampicillin treatment, although bacterial growth was slightly reduced at 1.0 μg mL^−1^, the unaltered growth at 0.1–0.5 μg mL^−1^ confirms that the observed increase in fluorescence is genuine and not a result of signal normalization (Supplementary Figure S5). Furthermore, neither tyrosol nor ampicillin treatment affected the fluorescence of the strain constitutively expressing mScarlet I under the control of the J23100 promoter, indicating that these chemicals do not interfere with the mScarlet I fluorophore (Supplementary Figure S6). Taken together, these results demonstrate that cdiGEBS is capable of reflecting the effects of typical chemicals on cellular c-di-GMP levels and even has the potential to be used for validating the binding affinities between various chemicals and c-di-GMP *in vivo*.

Recent studies have emphasized the strategy of developing anti-biofilm compounds by reducing cellular c-di-GMP levels for the treatment of biofilm-associated diseases (Martin et al., 2021; Hultqvist et al., 2024). The high sensitivity of our sensor suggests strong potential for its use as a high-throughput screening platform to identify novel agents that reduce intracellular c-di-GMP concentrations. Future efforts focusing on screening compounds that inhibit DGC activity, activate PDE activity, or sequester free c-di-GMP by combining tools of our sensor and fluorescence-activated cell sorting (FACS) could offer new insights for combating biofilm-associated diseases.

## 4 Discussion

In this work, we developed a c-di-GMP biosensor (cdiGEBS) based on the transcriptional activity of the c-di-GMP-responsive activator MrkH (Yang et al., 2013), achieving a high fluorescence dynamic range of 23-fold. The moderate expression of the sensor module MrkH using the medium-copy plasmid pBAD33 and the overexpression of the mScarlet I reporter with the high-copy plasmid pET28b(+) likely contribute to this high fluorescence dynamic range. Remarkably, our sensor demonstrates the capability in detecting subtle intracellular c-di-GMP changes, enabling it to be used to identify novel DGCs, distinguish various c-di-GMP synthesis activities of DGC mutants, as well as assess the effects of chemical agents on cellular c-di-GMP levels. However, while our sensor, based on TF activity, is well-suited for detecting steady-state differences in the c-di-GMP concentration, it is not designed for tracking rapid fluctuations of c-di-GMP. Consistent with this, approximately 5 h were required to observe significant fluorescence changes reflecting differences in c-di-GMP levels between the strain expressing the c-di-GMP synthesis enzyme WspR D70E and the strain that does not (Supplementary Figure S7).

Biofilm formation enhances bacterial resistance to antibiotics and the human immune system, making it closely associated with the development of urinary tract infections, cystic fibrosis lung infections, and other diseases (Gomez and Waters, 2023; Hultqvist et al., 2024). Studies have shown that elevated levels of intracellular c-di-GMP play a crucial role in the bacterial transition to a biofilm state (Jenal et al., 2017; Römling et al., 2013). Recent studies have emphasized the strategy of developing anti-biofilm compounds by reducing cellular c-di-GMP levels for the treatment of biofilm-associated diseases (Hultqvist et al., 2024; Martin et al., 2021). Although TF activity-based sensors could not provide cellular insights at the single-cell level, the high sensitivity of our sensor suggests strong potential for its use as a high-throughput screening platform to identify novel agents that reduce intracellular c-di-GMP concentrations. Considering the more complex c-di-GMP-mediated networks in pathogens such as *Pseudomonas aeruginosa* and *Vibrio cholerae* compared to *E. coli*, more studies are needed to determine whether cdiGEBS can effectively capture dynamic changes in c-di-GMP concentrations in these bacteria. Nevertheless, future efforts focusing on screening compounds that inhibit DGC activity, activate PDE activity, or sequester free c-di-GMP in *E. coli* by combining tools of our sensor and fluorescence-activated cell sorting (FACS) could offer new insights for combating biofilm-associated diseases.

In conclusion, we present a novel genetically encoded c-di-GMP biosensor, cdiGEBS, which not only enables more straightforward quantification of cellular c-di-GMP compared to traditional phenotypic assays including Congo red staining and crystal violet assays but also exceeds the fluorescence dynamic range of most existing biosensors, i.e., a 21-fold increase compared with mScarlet I-mVenus^NB^-MrkH and a 20-fold increase over CensYBL. The application of cdiGEBS in investigating DGC and PDE activities, as well as the effects of chemical agents on cellular c-di-GMP levels, underscores its potential for high-throughput screening of c-di-GMP-targeting agents, offering significant implications for biomedical applications.

## Data Availability

The original contributions presented in the study are included in the article/Supplementary Material, further inquiries can be directed to the corresponding authors.
