# Supplementary material for: A genetically encoded fluorescent biosensor for sensitive detection of cellular c-di-GMP levels in Escherichia coli
Source: Front Chem. 2025 Jan 10;12:1528626. doi: 10.3389/fchem.2024.1528626 (PMC11757272; doi:10.3389/fchem.2024.1528626)
Supplement: Supplementary file 1 [file DataSheet1.pdf]

Supplementary Information

A genetically encoded fluorescent biosensor for sensitive detection of cellular c-di-GMP levels in *Escherichia coli*

He Li[1], Shu Quan[1, 2*], Wei He[1, 3*]

Supplementary Files

A genetically encoded fluorescent biosensor for sensitive detection of cellular c-di-GMP levels in *Escherichia coli*

He Li[1], Shu Quan[1, 2*], Wei He[1, 3*]

[1]Shanghai Frontiers Science Center of Optogenetic Techniques for Cell Metabolism, East China University of Science and Technology, Shanghai 200237, China;

[2]State Key Laboratory of Microbial Metabolism, School of Life Sciences and Biotechnology, Zhangjiang Institute for Advanced Study, Shanghai Jiao Tong University, Shanghai 201203, China;

[3]State Key Laboratory of Molecular Biology, Shanghai Institute of Biochemistry and Cell Biology, Center for Excellence in Molecular Cell Science, Chinese Academy of Sciences, Shanghai 200031, China;

* Corresponding author.

E-mail address: shuquan@sjtu.edu.cn (S.Q.); whe@mail.ecust.edu.cn (W.H.)

Supplementary Figures

Contents：

Figure S1. Optimization of operating conditions for cdiGEBS

Figure S2. Expression level analysis of the wild-type and mutant form of the transcriptional regulator MrkH

Figure S3. Evaluation of the impact of L-arabinose on mScarlet I fluorescence

Figure S4. Expression level analysis of WspR wild type and mutants

Figure S5. Effects of CSP, tyrosol, and ampicillin on the growth of strains containing cdiGEBS

Figure S6. Effects of tyrosol and ampicillin on the mScarlet I fluorescence

Figure S7. Comparison of fluorescence signals between the strain expressing cdiGEBS and the strain co-expressing the biosensor and WspR D70E at different induction time points

Table S1. List of DNA oligonucleotides

Table S2. List of gene sequences

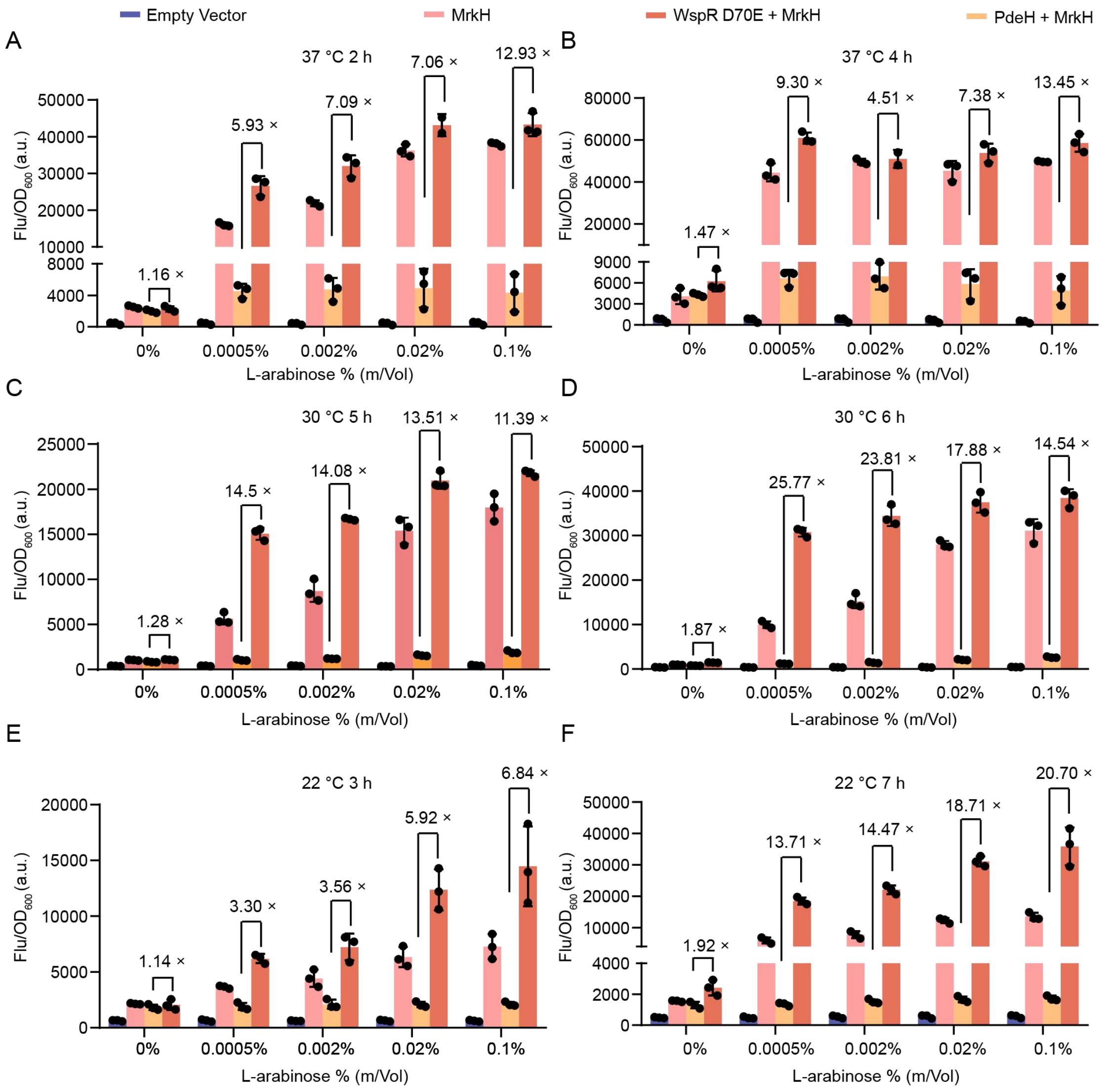

Figure S1. Optimization of operating conditions for cdiGEBS. (A-B) Fluorescence intensities of the strain harboring cidGEBS induced with 0% to 0.1% of L-arabinose at 37 °C for 2 h (A) and 4 h (B). (C-D) Fluorescence intensities of the strain harboring cidGEBS induced with 0% to 0.1% of L-arabinose at 30 °C for 5 h (C) and 6 h (D). (E-F) Fluorescence intensities of the strain harboring cidGEBS induced with 0% to 0.1% of L-arabinose at 22 °C for 3 h (E) and 7 h (F). The excitation wavelength is set at 540 ± 35 nm and the emission wavelength is set at 600 ± 40 nm for fluorescence measurement. Individual data points (black dots) and mean ± SD (n = 3) are shown.

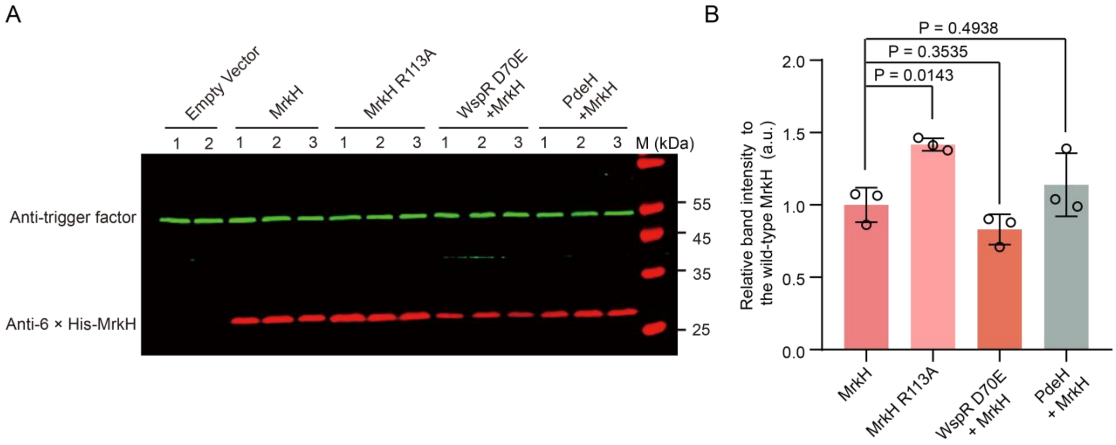

Figure S2. Expression level analysis of the wild-type and mutant form of the transcriptional regulator MrkH. (A) Immunoblotting of 6 × His-MrkH in the indicated samples using the antibody against 6 × His tag. A 6 × His tag was fused to the N-terminus of MrkH to facilitate immunoblotting and normalize band intensities. Trigger factor was used as a loading control to normalize sample loading. Results from three biological replicates are shown. (B) Quantification and comparison of the MrkH and MrkH R113A steady-state levels in the indicated strains according to the band intensity of western blots as shown in (A). Statistical analysis was performed with GraphPad Prism 8 using one-way ANOVA with Tukey's multiple comparisons tests.

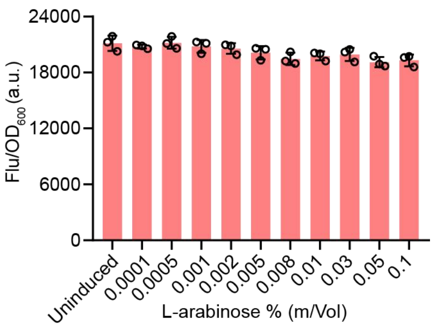

Figure S3. Evaluation of the impact of L-arabinose on mScarlet I fluorescence. The mScarlet I fluorescence intensities of strains expressing cdiGEBS in the presence of increasing concentrations of L-arabinose. Fluorescence was recorded by using a microplate reader with the excitation and emission wavelengths set at 540 ± 35 nm and 600 ± 40 nm, respectively. Individual data points (circles) and mean ± SD (n = 3) are shown.

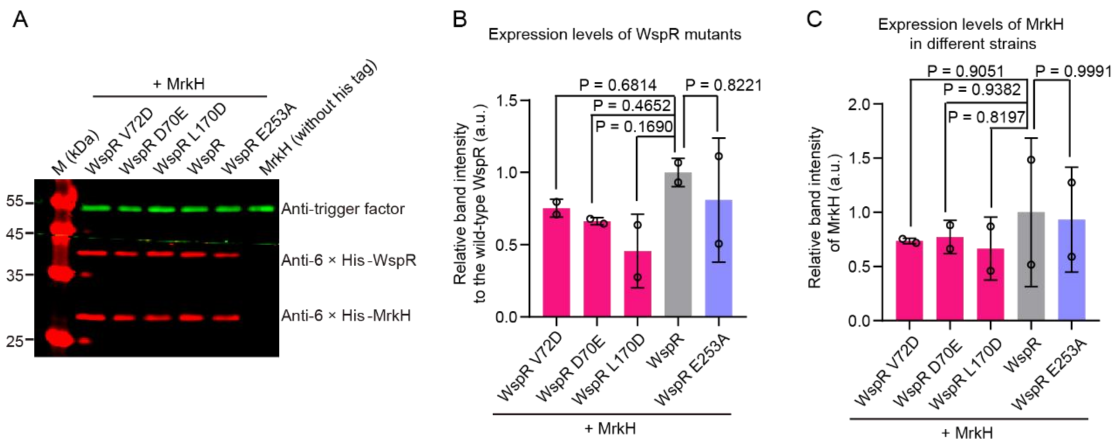

Figure S4. Expression level analysis of WspR wild type and mutants. (A) Immunoblotting of 6 × His-MrkH and 6 × His-WspR mutants in the indicated samples using the antibody against 6 × His tag. A 6 × His tag was fused to the N-terminus of MrkH and WspR for immunoblotting and band intensity normalization. Trigger factor was used as a loading control to normalize sample loading. A representative result of two biological replicates is shown. (B-C) Quantification and comparison of the steady-state levels of various WspR mutants (B) and MrkH (C) in the indicated strains based on the band intensity of western blots as shown in (A). Statistical analysis was performed with GraphPad Prism 8 using one-way ANOVA with Tukey's multiple comparisons tests.

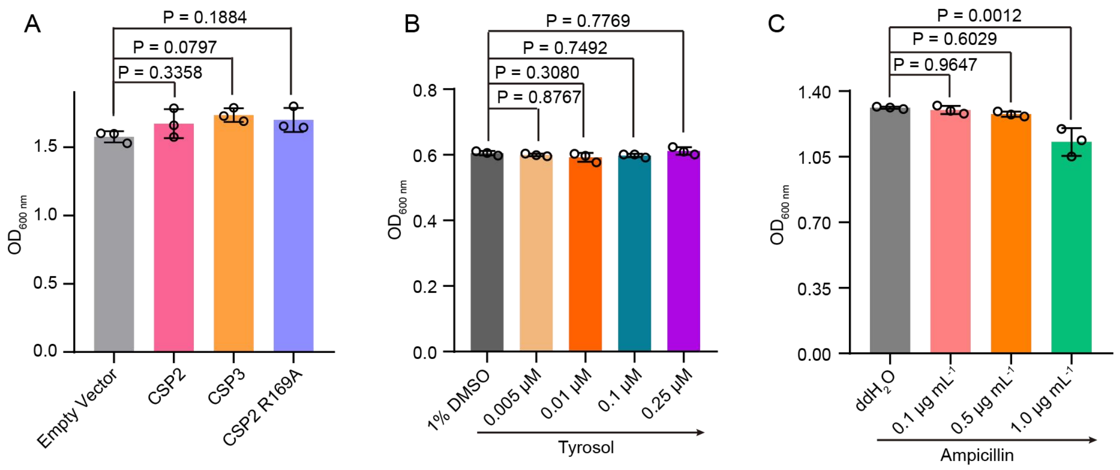

Figure S5. Effects of CSP, tyrosol, and ampicillin on the growth of strains containing cdiGEBS. (A) Cell densities ($OD_{600\ nm}$) of the strains co-expressing cdiGEBS and various CSPs after cultivation in 4 mL LB media for 9 h. (B-C) Cell densities ($OD_{600\ nm}$) of the strain harboring cdiGEBS after cultivation in a 96-well plate for 21 h in the presence of various concentrations of tyrosol (B) or ampicillin (C). Individual data points (circles) and mean ± SD (n = 3) are shown. Statistical analysis was performed with GraphPad Prism 8 using one-way ANOVA with Tukey's multiple comparisons tests.

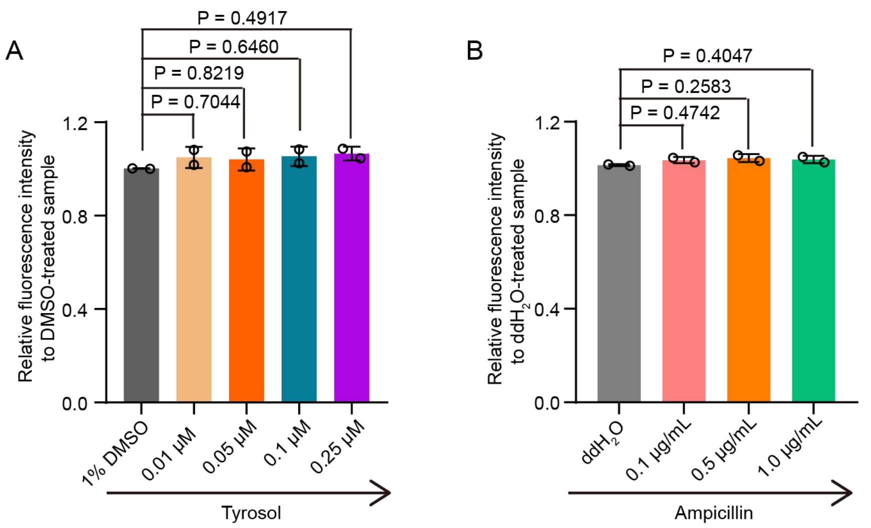

Figure S6. Effects of tyrosol and ampicillin on the mScarlet I fluorescence. (A-B) Fluorescence intensities of the strain constitutively expressing mScarlet I in the presence and absence of various concentrations of tyrosol (A) or ampicillin (B). Fluorescence was recorded by using a fluorescence spectrometer with the excitation and emission wavelengths set at 568 nm and 589 nm, respectively. Individual data points (circles) and mean ± SD (n = 2) are shown. Statistical analysis was performed with GraphPad Prism 8 using one-way ANOVA with Tukey's multiple comparisons tests.

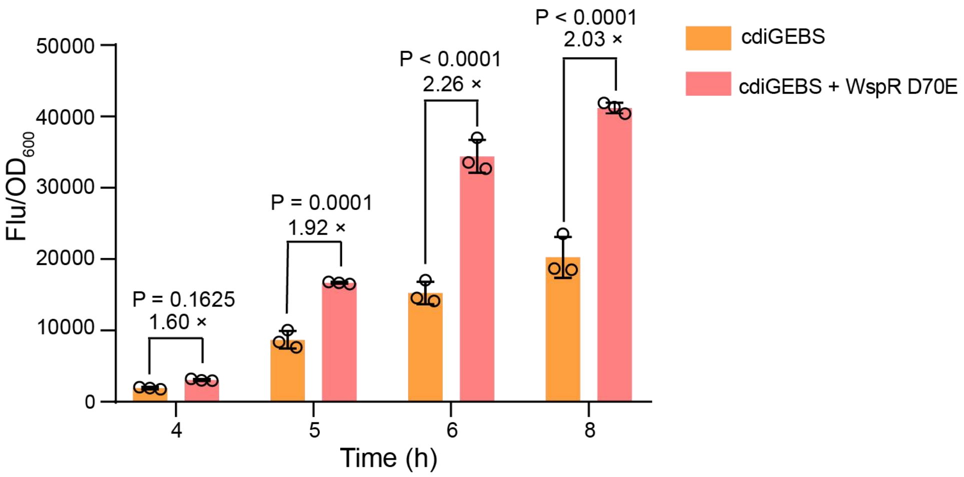

Figure S7. Comparison of fluorescence signals between the strain expressing cdiGEBS and the strain co-expressing the biosensor and WspR D70E at different induction time points. Samples were induced with 0.002% L-arabinose at 30 °C for 4, 5, 6, and 8 h. Fluorescence was recorded by using a microplate reader with the excitation and emission wavelengths set at 540 ± 35 nm and 600 ± 40 nm, respectively. Individual data points (circles) and mean ± SD (n = 3) are shown. Statistical analysis was performed with GraphPad Prism 8 using one-way ANOVA with Tukey's multiple comparisons tests.

Supplementary Tables

Supplementary Table S1. List of DNA oligonucleotides

Primer	Sequence
*mrkH*_FP	AGCGAATTCGAGCTCGGTACCAGGAGGAATTCATGACCG AGGGTACCATTAAGAC
*mrkH*_RP	AGCTTGCATGCCTGCAGGTCGACTCTAGATTAGATACGTT TCTTACGCTTCGC
*wspR D70E*_FP	AGCTTGCATGCCTGCAGGTCGACTCTAGATCAGCCCGCC GGGGCCGG
*wspR D70E*_RP	AGCTTGCATGCCTGCAGGTCGACTCTAGATCAGCCCGCC GGGGCCGG
*pdeH*_FP	AGCGTAAGAAACGTATCTAAGGAACAGACATGATAAGGC AGGTTATCCAGCG
*pdeH*_RP	TGCATGCCTGCAGGTCGACTCTAGATTATAGCGCCAGAA CCGCCG
*wspR D70A*_FP	AGCCGACGGTGATCCTCCAGGCGCTGGTGATGCCCGGCG TCGA
*wspR D70A*_RP	TCGACGCCGGGCATCACCAGCGCCTGGAGGATCACCGTC GGCT
*wspR*_FP	AGATCAAGCCGACGGTGATCCTCCAGGATCTGGTGATGC CCGGCGTCGACGG
*wspR*_RP	CCGTCGACGCCGGGCATCACCAGATCCTGGAGGATCACC GTCGGCTTGATCT
*wspR L170D*_FP	GAGACCAACCTGGTGCTGCAGCGGGATATGAACTCCGAC GGCCTGACCGGGCT
*wspR L170D*_RP	AGCCCGGTCAGGCCGTCGGAGTTCATATCCCGCTGCAGC ACCAGGTTGGTCTC
*wspR V72D*_FP	AGATCAAGCCGACGGTGATCCTCCAGGATCTGGACATGC

	CCGGCGTCGACGGCCTCACG
*wspR V72D*_RP	CGTGAGGCCGTCGACGCCGGGCATGTCCAGATCCTGGAGGATCACCGTCGGCTTGATCT
*dgcF*_FP	GAAGCGTAAGAAACGTATCTAAGGAACAGACATGCTTGCGGACTGGTTCAGTG
*dgcF*_RP	TGCATGCCTGCAGGTCGACTCTAGATCAGACAACTTCCTCACCGTAAC
*dgcT*_FP	AGCGTAAGAAACGTATCTAAGGAACAGACATGGAAAAAGACTATTTGAGAATTAGTAG
*dgcT*_RP	TGCATGCCTGCAGGTCGACTCTAGATTATGGTGACTCACAAAAATTCACCAC
*dgcI*_FP	GAAGCGTAAGAAACGTATCTAAGGAACAGACATGTCCAGAATCAATAAGTTCGTAC
*dgcI*_RP	TGCATGCCTGCAGGTCGACTCTAGATTACTGCCAGCTAATCTGTGCC
*pdeK*_FP	GAAGCGTAAGAAACGTATCTAAGGAACAGACATGCGCGTAAGTCGCTCGTTAAC
*pdeK*_RP	TGCATGCCTGCAGGTCGACTCTAGACTACTTTTCTTCCAGGTAACTCTCTTC
*dgcF E224A*_FP	TGGTGGCGCGAATGGGCGGCGCGGAATTTGCTGTTGCAGTGCCGTCGGTGAATC
*dgcF E224A*_RP	GATTCACCGACGGCACTGCAACAGCAAATTCCGCGCCGCCCATTCGCGCCACCA
*dgcT E361A*_FP	ACCTTTTAGCGCGCGTCGGCGGCGCGGAGTTTGGCGTCTTGCTGACGGACATCGATAC
*dgcT E361A*_RP	GTATCGATGTCCGTCAGCAAGACGCCAAACTCCGCGCCGCCGACGCGCGCTAAAAGGT
proC promoter-*mrkH*_FP	TGTCGGTGAACGCTCTCCTGAGTAGGACAAATCACAGCTAACACCACGTCGTC

proC promoter-*mrkH*_RP	CAACGTTCAAATCCGCTCCCGGCGGATAAAACGAAAGGC CCAGTCTTTC
pBAD33_FP	ATTTGTCCTACTCAGGAGAGCGTTCAC
pBAD33_RP	CCGCCGGGAGCGGATTTGAACCCGCCGGGAGCGGATTT GAAC

Supplementary Table S2. List of gene sequences

Promoter or gene	Sequence
mrkApro	GCTGCGCTGTAAACAACCACCCTCGCGTTTTCATCTATCAATG GCTGTTTATTAATAGTCGATGGTTATCTGTTATATAACTTAATG AAACGTGAACAAATGTATATTTGTCGGCGAATAAATAGCATTC TTTGACGCCGATAGCACCAG
proC	CACAGCTAACACCACGTCGTCCCTATCTGCTGCCCTAGGTCTA TGAGTGGTTGCTGGATAACTTTACGGGCATGCATAAGGCTCGT ATGATATATTCAGGGAGACCACAACGGTTTCCCTCTACAAATA ATTTTGTTTAACTTT
mrkH	ATGACCGAGGGTACCATTAAGACCAGCAAATACGAAATCATT GCGATCTTCCGTGAGGAACTGCGTAAGCGTACCGAGATCGAA ATTTTCTTTAACAACACCAGCATCATCACCCAGCTGACCCGTG TGGATTTCGCGGAGTTTCACATCCAAACCCACCGTAAGATTC CGAGCGGTCACAAAATCCGTTTCCTGCTGCACAGCGACAGC GGCAAAATTGAGTTTAACGCGGCGCTGACCAAGCACGATAA CAGCGGTGTTGACAAAGGCATCCGTTACGCGTTTAGCCTGCC GGAATGCCTGCAGGTGGTTCAACGTCGTCGTGATCCGCGTTT CCGTCTGCGTCACGAGCACGACTTTTACTGCCGTGGTCGTCA CAAGAACGGCGAAAACTACCTGTTCGACATCAAAGATATTAG CGACGGTGGCTGCGCGCTGATGACCAAGACCCCGAACCTGA AATTTCTGAGCCACAACGCGCTGCTGAAGAACGCGGTGCTGA TGCTGGCGGAGTATGGCGAAATCACCATTGATCTGGTGGTTA AAAACGTGATCGTTATTACCCTGGACAACGCGAACGAGGAA AGCGAAAGCTACTATCAGATCAGCTGCCAATTCAAGTTTCGT CACCTGGACGATCAGCGTCGTATCGAGAAGATTCTGCTGGAT CTGATTCTGGAAGCGAAGCGTAAGAAACGTATCTAA
mScarlet I	ATGAGTAAAGGAGAAGCTGTGATTAAAGAGTTCATGCGCTTC AAAGTTCACATGGAGGGTTCTATGAACGGTCACGAGTTCGAG

	ATCGAAGGCGAAGGCGAGGGCCGTCCGTATGAAGGCACCCA
	GACCGCCAAACTGAAAGTGACTAAAGGCGGCCCGCTGCCTT
	TTTCCTGGGACATCCTGAGCCCGCAATTTATGTACGGTTCTAG
	GGCGTTCATCAAACACCCAGCGGATATCCCGGACTATTATAAG
	CAGTCTTTTCCGGAAGGTTTCAAGTGGGAACGCGTAATGAAT
	TTTGAAGATGGTGGTGCCGTGACCGTCACTCAGGACACCTCC
	CTGGAGGATGGCACCCTGATCTATAAAGTTAAACTGCGTGGT
	ACTAATTTTCCACCTGATGGCCCGGTGATGCAGAAAAAGACG
	ATGGGTTGGGAGGCGTCTACCGAACGCTTGTATCCGGAAGAT
	GGTGTGCTGAAAGGCGACATTAAAATGGCCCTGCGCCTGAA
	AGATGGCGGCCGCTATCTGGCTGACTTCAAAACCACGTATAA
	AGCCAAGAAACCTGTGCAGATGCCTGGCGCGTACAATGTGG
	ACCGCAAACTGGACATCACCTCTCATAATGAAGATTATACGGT
	GGTAGAGCAATATGAGCGCTCCGAGGGTCGTCATTCTACCGG
	TGGCATGGATGAACTATACAAATAAATGAGTAAAGGAGAAGC
	TGTGATTAAAGAGTTCATGCGCTTCAAAGTTCACATGGAGGG
	TTCTATGAACGGTCACGAGTTCGAGATCGAAGGCGAAGGCG
	AGGGCCGTCCGTATGAAGGCACCCAGACCGCCAAACTGAAA
	GTGACTAAAGGCGGCCCGCTGCCTTTTTCCTGGGACATCCTG
	AGCCCGCAATTTATGTACGGTTCTAGGGCGTTCATCAAACACC
	CAGCGGATATCCCGGACTATTATAAGCAGTCTTTTCCGGAAGG
	TTTCAAGTGGGAACGCGTAATGAATTTTGAAGATGGTGGTGC
	CGTGACCGTCACTCAGGACACCTCCCTGGAGGATGGCACCCT
	GATCTATAAAGTTAAACTGCGTGGTACTAATTTTCCACCTGAT
	GGCCCGGTGATGCAGAAAAAGACGATGGGTTGGGAGGCGTC
	TACCGAACGCTTGTATCCGGAAGATGGTGTGCTGAAAGGCGA
	CATTAAAATGGCCCTGCGCCTGAAAGATGGCGGCCGCTATCT
	GGCTGACTTCAAAACCACGTATAAAGCCAAGAAACCTGTGC
	AGATGCCTGGCGCGTACAATGTGGACCGCAAACTGGACATCA

	CCTCTCATAATGAAGATTATACGGTGGTAGAGCAATATGAGCG<br>CTCCGAGGGTCGTCATTCTACCGGTGGCATGGATGAACTATAC<br>AAATAA
wspR D70E	ATGCACAACCCTCATGAGAGCAAGACCGACCTGGGCGCCCC<br>TCTGGACGGCGCGGTCATGGTACTGCTTGTCGATGATCAGGC<br>CATGATCGGAGAGGCTGTGCGTCGCTCACTGGCGAGCGAGG<br>CGGGCATCGACTTCCATTTCTGTTCCGATCCGCAGCAGGCGG<br>TGGCGGTGGCCAACCAGATCAAGCCGACGGTGATCCTCCAG<br>GAACTGGTGATGCCCGGCGTCGACGGCCTCACGCTGCTCGCT<br>GCCTACCGCGGCAACCCGGCGACCCGCGACATCCCGATCATC<br>GTCCTGTCGACCAAGGAAGAGCCGACGGTGAAGAGCGCGGC<br>GTTCGCCGCCGGCGCCAACGACTACCTGGTCAAGCTGCCGG<br>ACGCCATCGAGCTGGTGGCGCGGATCCGCTACCACTCGCGTT<br>CGTACATCGCCCTGCAGCAACGCGACGAGGCCTATCGGGCGC<br>TGCGCGAGAGCCAGCAGCAGTTGCTGGAGACCAACCTGGTG<br>CTGCAGCGGCTGATGAACTCCGACGGCCTGACCGGGCTCTCC<br>AACCGTCGTCACTTCGACGAATACCTGGAGATGGAGTGGCGC<br>CGCTCGCTGCGCGAGCAGTCGCAACTGTCGCTGCTGATGATC<br>GACGTGGACTATTTCAAGAGCTACAACGACACCTTCGGCCAC<br>GTCGCCGGCGACGAGGCCCTGCGCCAGGTCGCCGGGGCCAT<br>CCGCGAGGGCTGCAGTCGCTCCTCGGACCTGGCGGCTCGGT<br>ACGGCGGCGAGGAGTTCGCCATGGTCCTCCCGGGCACCTCG<br>CCGGGCGGCGCACGGTTGCTGGCGGAGAAGGTCCGGCGCAC<br>GGTGGAGAGCCTGCAGATCAGCCACGACCAGCCACGCCCGG<br>GCTCGCACCTGACCGTCAGCATCGGCGTCAGCACCCTGGTGC<br>CGGGCGGCGGCGGGCAAACCTTCCGGGTTCTCATCGAGATG<br>GCCGACCAGGCGCTCTACCAGGCCAAGAACAATGGTCGCAA<br>CCAGGTGGGCCTGATGGAACAGCCGGTGTCGCCGGCCCCGG<br>CGGGCTGA

pdeH	ATGATAAGGCAGGTTATCCAGCGAATAAGCAACCCTGAAGCA<br>AGCATCGAGAGCTTGCAGGAACGGCGTTTTTGGTTGCAGTGT<br>GAGCGTGCTTACACCTGGCAGCCGATCTATCAAACATGCGGG<br>CGGTTAATGGCCGTGGAGCTATTAACGGTGGTCACGCATCCC<br>TTGAACCCTTCGCAACGCCTGCCGCCGGATCGCTATTTTACTG<br>AAATCACCGTCAGCCATCGGATGGAGGTTGTGAAAGAGCAG<br>ATTGATTTGCTGGCGCAAAAAGCCGACTTCTTTATAGAGCAC<br>GGCCTGCTGGCATCGGTCAATATTGATGGCCCTACGCTCATCG<br>CCCTGCGTCAGCAACCAAAAATCCTGCGCCAGATTGAGCGTC<br>TTCCCTGGCTGCGTTTCGAACTGGTGGAGCATATCCGTCTGCC<br>GAAAGATTCAACCTTTGCCTCGATGTGTGAATTTGGCCCGCT<br>GTGGCTGGATGATTTTGGTACCGGGATGGCAAATTTCTCTGCG<br>CTAAGTGAAGTGCGTTATGACTACATCAAAATCGCGCGAGAA<br>CTGTTTGTGATGCTGCGTCAGTCGCCGGAAGGACGCACACTC<br>TTTTCTCAGCTTTTACATCTAATGAATCGCTATTGTCGCGGGGT<br>GATTGTCGAGGGCGTAGAAACGCCGGAAGAGTGGCGTGATG<br>TTCAGAACTCGCCCGCATTCGCCGCACAAGGCTGGTTTCTTT<br>CACGCCCGGCACCGATAGAAACGCTGAATACGGCGGTTCTGG<br>CGCTATAA
6 × his-mbp-csp2	ATGCATCATCATCATCATCACAGCAGCGGCCTGGTGCCGCGC<br>GGCAGCCATATGGCTAGCATGAAAATCGAAGAAGGTAAACTG<br>GTAATCTGGATTAACGGCGATAAAGGCTATAACGGTCTCGCTG<br>AAGTCGGTAAGAAATTCGAGAAAGATACCGGAATTAAAGTCA<br>CCGTTGAGCATCCGGATAAACTGGAAGAGAAATTCCCACAGG<br>TTGCGGCAACTGGCGATGGCCCTGACATTATCTTCTGGGCAC<br>ACGACCGCTTTGGTGGCTACGCTCAATCTGGCCTGTTGGCTG<br>AAATCACCCCGGACAAAGCGTTCCAGGACAAGCTGTATCCGT<br>TTACCTGGGATGCCGTACGTTACAACGGCAAGCTGATTGCTT<br>ACCCGATCGCTGTTGAAGCGTTATCGCTGATTTATAACAAAGA

	TCTGCTGCCGAACCCGCCAAAAACCTGGGAAGAGATCCCGG<br>CGCTGGATAAAGAACTGAAAGCGAAAGGTAAGAGCGCGCTG<br>ATGTTCAACCTGCAAGAACCGTACTTCACCTGGCCGCTGATT<br>GCTGCTGACGGGGGTTATGCGTTCAAGTATGAAAACGGCAAG<br>TACGACATTAAAGACGTGGGCGTGGATAACGCTGGCGCGAA<br>AGCGGGTCTGACCTTCCTGGTTGACCTGATTAAAAACAAACA<br>CATGAATGCAGACACCGATTACTCCATCGCAGAAGCTGCCTT<br>TAATAAAGGCGAAACAGCGATGACCATCAACGGCCCGTGGG<br>CATGGTCCAACATCGACACCAGCAAAGTGAATTATGGTGTAA<br>CGGTACTGCCGACCTTCAAGGGTCAACCATCCAAACCGTTCG<br>TTGGCGTGCTGAGCGCAGGTATTAACGCCGCCAGTCCGAACA<br>AAGAGCTGGCGAAAGAGTTCCTCGAAAACTATCTGCTGACT<br>GATGAAGGTCTGGAAGCGGTTAATAAAGACAAACCGCTGGG<br>TGCCGTAGCGCTGAAGTCTTACGAGGAAGAGTTGGCGAAAG<br>ATCCACGTATTGCCGCCACCATGGAAAACGCCCAGAAAGGTG<br>AAATCATGCCGAACATCCCGCAGATGTCCGCTTTCTGGTATGC<br>CGTGCGTACTGCGGTGATCAACGCCGCCAGCGGTCGTCAGAC<br>TGTCGATGAAGCCCTGAAAGACGCGCAGACTAGCAAGCCGC<br>GCGAATGGGTCGAGGCCGTCGCCTATGTCGGGCCTGATCGCC<br>GTCGTTTCAACTCGGCGGACTATAAGGGGCCGCGCCGCCGCA<br>AGGCGGACGCCAGCTAA
6 × his-mbp-csp2 R169A	ATGCATCATCATCATCATCACAGCAGCGGCCTGGTGCCGCGC<br>GGCAGCCATATGGCTAGCATGAAAATCGAAGAAGGTAAACTG<br>GTAATCTGGATTAACGGCGATAAAGGCTATAACGGTCTCGCTG<br>AAGTCGGTAAGAAATTCGAGAAAGATACCGGAATTAAAGTCA<br>CCGTTGAGCATCCGGATAAACTGGAAGAGAAATTCCCACAGG<br>TTGCGGCAACTGGCGATGGCCCTGACATTATCTTCTGGGCAC<br>ACGACCGCTTTGGTGGCTACGCTCAATCTGGCCTGTTGGCTG<br>AAATCACCCCGGACAAAGCGTTCCAGGACAAGCTGTATCCGT

	TTACCTGGGATGCCGTACGTTACAACGGCAAGCTGATTGCTT<br>ACCCGATCGCTGTTGAAGCGTTATCGCTGATTTATAACAAAGA<br>TCTGCTGCCGAACCCGCCAAAAACCTGGGAAGAGATCCCGG<br>CGCTGGATAAAGAACTGAAAGCGAAAGGTAAGAGCGCGCTG<br>ATGTTCAACCTGCAAGAACCGTACTTCACCTGGCCGCTGATT<br>GCTGCTGACGGGGGTTATGCGTTCAAGTATGAAAACGGCAAG<br>TACGACATTAAAGACGTGGGCGTGGATAACGCTGGCGCGAA<br>AGCGGGTCTGACCTTCCTGGTTGACCTGATTAAAAACAAACA<br>CATGAATGCAGACACCGATTACTCCATCGCAGAAGCTGCCTT<br>TAATAAAGGCGAAACAGCGATGACCATCAACGGCCCGTGGG<br>CATGGTCCAACATCGACACCAGCAAAGTGAATTATGGTGTAA<br>CGGTACTGCCGACCTTCAAGGGTCAACCATCCAAACCGTTCG<br>TTGGCGTGCTGAGCGCAGGTATTAACGCCGCCAGTCCGAACA<br>AAGAGCTGGCGAAAGAGTTCCTCGAAAACTATCTGCTGACT<br>GATGAAGGTCTGGAAGCGGTTAATAAAGACAAACCGCTGGG<br>TGCCGTAGCGCTGAAGTCTTACGAGGAAGAGTTGGCGAAAG<br>ATCCACGTATTGCCGCCACCATGGAAAACGCCCAGAAAGGTG<br>AAATCATGCCGAACATCCCGCAGATGTCCGCTTTCTGGTATGC<br>CGTGCGTACTGCGGTGATCAACGCCGCCAGCGGTCGTCAGAC<br>TGTCGATGAAGCCCTGAAAGACGCGCAGACTAGCAAGCCGC<br>GCGAATGGGTCGAGGCCGTCGCCTATGTCGGGCCTGATCGCC<br>GTCGTTTCAACTCGGCGGACTATAAGGGGCCGCGCCGCGCGA<br>AGGCGGACGCCAGCTAA
6 ×his-mbp-csp3	ATGCATCATCATCATCATCACAGCAGCGGCCTGGTGCCGCGC<br>GGCAGCCATATGGCTAGCATGAAAATCGAAGAAGGTAAACTG<br>GTAATCTGGATTAACGGCGATAAAGGCTATAACGGTCTCGCTG<br>AAGTCGGTAAGAAATTCGAGAAAGATACCGGAATTAAAGTCA<br>CCGTTGAGCATCCGGATAAACTGGAAGAGAAATTCCCACAGG<br>TTGCGGCAACTGGCGATGGCCCTGACATTATCTTCTGGGCAC

	ACGACCGCTTTGGTGGCTACGCTCAATCTGGCCTGTTGGCTG AAATCACCCCGGACAAAGCGTTCCAGGACAAGCTGTATCCGT TTACCTGGGATGCCGTACGTTACAACGGCAAGCTGATTGCTT ACCCGATCGCTGTTGAAGCGTTATCGCTGATTTATAACAAAGA TCTGCTGCCGAACCCGCCAAAAACCTGGGAAGAGATCCCGG CGCTGGATAAAGAACTGAAAGCGAAAGGTAAGAGCGCGCTG ATGTTCAACCTGCAAGAACCGTACTTCACCTGGCCGCTGATT GCTGCTGACGGGGGTTATGCGTTCAAGTATGAAAACGGCAAG TACGACATTAAAGACGTGGGCGTGGATAACGCTGGCGCGAA AGCGGGTCTGACCTTCCTGGTTGACCTGATTAAAAACAAACA CATGAATGCAGACACCGATTACTCCATCGCAGAAGCTGCCTT TAATAAAGGCGAAACAGCGATGACCATCAACGGCCCGTGGG CATGGTCCAACATCGACACCAGCAAAGTGAATTATGGTGTAA CGGTACTGCCGACCTTCAAGGGTCAACCATCCAAACCGTTCG TTGGCGTGCTGAGCGCAGGTATTAACGCCGCCAGTCCGAACA AAGAGCTGGCGAAAGAGTTCCTCGAAAACTATCTGCTGACT GATGAAGGTCTGGAAGCGGTTAATAAAGACAAACCGCTGGG TGCCGTAGCGCTGAAGTCTTACGAGGAAGAGTTGGCGAAAG ATCCACGTATTGCCGCCACCATGGAAAACGCCCAGAAAGGTG AAATCATGCCGAACATCCCGCAGATGTCCGCTTTCTGGTATGC CGTGCGTACTGCGGTGATCAACGCCGCCAGCGGTCGTCAGAC TGTCGATGAAGCCCTGAAAGACGCGCAGACTTGGGTCGAGG CCGTCGCCTATGTCGGGCCTGATCGCCGTCGTTTCAACTCGG CGGACTATAAGGGGCCGCGCCGCCGCAAGGCGGACGCCTAA
